# Sexually dimorphic effects of a prenatal immune challenge on social play and vasopressin expression in juvenile rats

**DOI:** 10.1186/2042-6410-3-15

**Published:** 2012-06-14

**Authors:** Patrick V Taylor, Alexa H Veenema, Matthew J Paul, Remco Bredewold, Stephanie Isaacs, Geert J de Vries

**Affiliations:** 1 Center for Neuroendocrine Studies and Department of Psychology, University of Massachusetts, Amherst, MA, 01003, USA; 2 Department of Psychology, Boston College, Chestnut Hill, MA, 02467, USA

**Keywords:** Lipopolysaccharides, Bed nucleus of the stria terminalis, Medial amygdaloid nucleus, Prenatal, Play behavior, Sex differences, SDN-POA, Development

## Abstract

**Background:**

Infectious diseases and inflammation during pregnancy increase the offspring’s risk for behavioral disorders. However, how immune stress affects neural circuitry during development is not well known. We tested whether a prenatal immune challenge interferes with the development of social play and with neural circuits implicated in social behavior.

**Methods:**

Pregnant rats were given intraperitoneal injections of the bacterial endotoxin lipopolysaccharide (LPS – 100 μg /kg) or saline on the 15th day of pregnancy. Offspring were tested for social play behaviors between postnatal days 26–40. Brains were harvested on postnatal day 45 and processed for arginine vasopressin (AVP) mRNA *in situ* hybridization.

**Results:**

In males, LPS treatment reduced the frequency of juvenile play behavior and reduced AVP mRNA expression in the medial amygdala and bed nucleus of the stria terminalis. These effects were not found in females. LPS treatment did not change AVP mRNA expression in the suprachiasmatic nucleus, paraventricular nucleus, or supraoptic nucleus of either sex, nor did it affect the sex difference in the size of the sexually dimorphic nucleus of the preoptic area.

**Conclusions:**

Given AVP’s central role in regulating social behavior, the sexually dimorphic effects of prenatal LPS treatment on male AVP mRNA expression may contribute to the sexually dimorphic effect of LPS on male social play and may, therefore, increase understanding of factors that contribute to sex differences in social psychopathology.

## Background

Children of mothers who were afflicted by an infectious disease during pregnancy have a higher risk for schizophrenia, autism spectrum disorders, mental retardation, and other mental disorders [1,2]. Animal models used to study the effects of infectious disease during development often use lipopolysaccharide (LPS), a non-infectious bacterial antigen derived from the cell wall of gram negative bacteria to activate the immune system [3]. For example, mice whose mothers were treated with LPS during pregnancy show less aggression and more social grooming behavior in adulthood [4]. Remarkably, although many disorders of social behavior emerge during childhood, very few studies have addressed the effects of prenatal immune activation on social behavior during development.

We hypothesized that prenatal immune activation alters juvenile social play behavior just as it alters adult behavior, and that it does so by changing neural circuitry involved in social behavior. We focused on social play, which in rats is the primary social behavior performed during pre-pubertal life [5], and on the AVP innervation of the brain, as this system (or its non-mammalian homologue vasotocin innervation) has been implicated in a wide variety of social behaviors across a broad range of species [6,7]. Moreover, blocking AVP receptors centrally reduces social play in 35-day-old male rats while increasing it in females [8], and postnatal stressors that affect social play in rats modify AVP expression in the paraventricular nucleus (PVN) and supraoptic nucleus (SON) [9]. Given that developmental perturbations, including LPS treatment, often affect males and females differently [10,11], and that AVP innervation is highly sexually dimorphic in rats [12], we compare the effects of LPS on both sexes. We find that prenatal LPS exposure reduces juvenile play behavior and AVP mRNA expression in the medial amygdaloid nucleus (MeA) as well as the bed nucleus of the stria terminalis (BST) in male but not in female rats. Since the MeA is known to be important for normal levels of social play behavior in males but not in females [13], these results suggest a way in which prenatal immune activation may differentially affect the development of social behavior in males and females.

## Methods

### Animals

Adult Wistar rats were obtained from Charles River, Wilmington, MA, and paired for mating. The day a sperm plug was found was defined as embryonic day 0 (E0). Males were removed that day, and females were housed individually in standard rat cages under a 14:10 light:dark cycle with lights off at 10 am. Temperature was maintained at 22 °C. Food and water were provided *ad libitum*. Animals were not maintained under specified pathogen free (SPF) conditions. All procedures were conducted in accordance with the NIH Guide for the Care and Use of Laboratory Animals and approved by the Institutional Animal Care and Use Committee.

### Experimental design

On E15, dams were injected intraperitoneally with 100 μg/kg LPS (E. coli O26:B6; Sigma-Aldrich, St. Louis, MO) or sterile saline. LPS was chosen as it is one of the most commonly used immune stimulants, for which many of the cellular inflammatory cascades have been worked out; the dose of 100 μg/kg of LPS is commonly used in studies of prenatal effects of immune stimulation [14]. Following injection, the dams were monitored twice daily for two days to check for overt signs of sickness as defined by the National Research Council’s 1996 *Guide for the Care and Use of Laboratory Animals*. Within one day after birth, litters were culled to produce litter sizes between four and six pups, and the number of males and females was kept equal across treatment. Litters were weaned at 22 days of age (day of birth being day 0) and housed in sex-mixed groups at 25 days of age, resulting in seven saline litters and five LPS litters. Behavior of offspring was digitally video-recorded on five different days between 26 to 40 days of age. Animals were weighed at 35 and 45 days of age. At 45 days of age, all offspring were killed, their brains removed and snap-frozen in 2-methyl-butane on dry ice. Brains were stored at −80 °C. For ease of description, animals from LPS treated-dams will be called ‘LPS Males’ and ‘LPS Females,’ the control animals will be called ‘Saline Males’ and ‘Saline Females.’

### Play behavior

Play behavior was assessed at the beginning of the dark phase. Rats were habituated to the testing condition by moving the home cage into the testing room for three hours on two consecutive days prior to the tests. All animals were tested under two different conditions on the following days: (1) in treatment-matched pairs (LPS pairs and saline pairs at 26, 37, and 42 days of age), and (2) treatment-mixed pairs (LPS/saline pairs at 30 and 40 days of age). Rats were housed individually in a new cage for 1 h before being paired with a sex- and age-matched rat. Treatment-matched pairs came from the same home cage. After recording behavior for 10 min, rats were returned to their home cage. At 35 days of age, 22 rats were sacrificed, and their brains set aside for future analysis. A researcher blind to the treatment conditions used JWatcher software (http://www.jwatcher.ucla.edu) to score the frequency of Boxing & Wrestling, Pinning, and Pouncing, as defined in [15]. Total Play was calculated as the total of these three frequencies.

#### *In situ* hybridization

Each brain was cut transversally at 12 μm into three series, thaw-mounted onto Colorfrost/plus slides (Thermo Fisher Scientific, Pittsburgh, PA), and stored at −80 °C. One series of sections was postfixed in 4 % paraformaldehyde for 5 min and rinsed in 0.1 M phosphate-buffered saline (pH 7.4) for 2 min, both solutions at 4 °C. *In situ* hybridization was performed as published previously [16,17] using a mixture of two oligodeoxyribose antisense probes. Probe 1 and 2 are complementary to the regions that code for amino acids 127–141 and 143–159 of the AVP prohormone, which are in the glycopeptide region near the COOH-terminal. The probes were labeled at the 3’ end with 35 S-dATP (PerkinElmer, Waltham, MA) using terminal deoxynucleotidyl transferase (Life Technologies Inc., Gaithersburg, MD). To locate the hybridization signal, slides were dipped in Kodak NTB-3 emulsion under safelight and stored desiccated in light tight boxes at 4 °C. After four weeks, slides were developed with Kodak D19 developer (1:1 with purified water) and fixed with Kodak Rapid Fix. Slides were rinsed in purified water, lightly counterstained with 2 % methyl green, dehydrated with 50 % ethanol, and coverslipped with Cytoseal 60 (Richard-Allen Scientific, Kalamazoo, MI).

### AVP mRNA analysis

For analysis of sections processed for AVP mRNA *in situ* hybridization, cells with a density of silver grains above background were counted in every third section by two observers blind to the treatment. Labeled cells in the bed nucleus of the stria terminalis (BST) and medial amygdaloid nucleus (MeA) were identified under dark-field illumination using a 20X objective and counted only if brightfield microscopy confirmed a methyl green-stained nucleus underneath the silver grains. As crowding of labeled cells prevented counting individual cells in the suprachiasmatic nucleus (SCN), supraoptic nucleus (SON), and paraventricular nucleus (PVN), AVP mRNA expression in these nuclei was digitally photographed throughout their rostro-caudal extent using a 4X objective under bright-field illumination. For each nucleus, we determined the total area above background and the integrated optical density (calculated as total area above background times average gray value (0–255) of thresholded pixels) using Image J software (NIH, Bethesda, MD).

### Volume of the sexually dimorphic nucleus of the preoptic area

As LPS treatment blunted the sex difference in AVP mRNA expression in the BST and MeA, we tested whether LPS had general effects on sexual differentiation by measuring the volume of the sexually dimorphic nucleus of the preoptic area (SDN-POA) [18]. Sections at the level of the SDN-POA from the second series of slides were thawed and allowed to dry at room temperature for 10 min, delipidated with a graded ethanol series and thionin-stained. Sections were coverslipped using permount (SP15-500, Fisher Scientific). Each section containing the SDN-POA was digitally photographed throughout the rostro-caudal extent of the nucleus using a 4X objective under bright-field illumination. An experimenter blind to the treatment traced and measured the area throughout the SDN-POA using Image J software (NIH, Bethesda, MD). Volume was calculated as the sum of area x 3 x 12 μm.

### Statistical analysis

To determine whether play behaviors differed significantly across age, paired t-tests were conducted on each behavioral measure (Total Play, Boxing & Wrestling, Pinning, and Pouncing) for all pairwise age combinations in matched pairs (P26 *vs.* P37, P37 *vs.* P42, and P26 *vs.* P42) and Mixed Pairs (P30 *vs.* P40). Out of these 24 comparisons, only one differed significantly between age groups (Pouncing at P26 *vs.* P37, p < 0.05). Because there was no systematic effect of age, all further behavioral analyses were based on individuals’ mean score (mean Total Play, mean Boxing & Wrestling, *etc.*). To avoid litter effects, behavioral scores and neural measures for males and females were averaged by litter and then analyzed using a two-way ANOVA (Sex X Treatment), see ref [19]. Planned comparisons (Fisher’s PLSD) were performed to evaluate 1) sex differences (Saline Males *versus* Saline Females), 2) LPS effects in males (LPS Males *versus* Saline Males), and 3) LPS effects in females (LPS Females *versus* Saline Females). Analyses were conducted using Statview 5.0.1 (SAS Institute Inc., Cary, NC).

## Results

### General effects of LPS exposure

Dams injected on embryonic day 15 showed increased red lachrymal secretions for one day following LPS injection, but no other overt signs of sickness were observed, and pregnancies were not aborted. LPS did not affect litter size or sex ratio, and pups showed no gross abnormalities.

### Development of social play behavior

**Figure 1:**
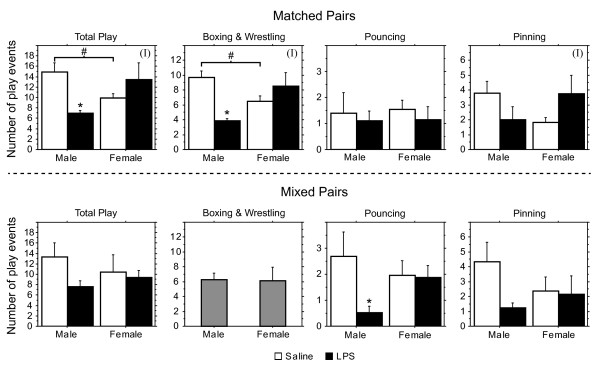
**Effect of prenatal LPS on play behavior.** Means + SEMs of the number of play events displayed in a ten-minute testing period. (I): in treatment-matched pairs, there was a significant interaction of treatment X sex for Total Play (ANOVA, *p* < 0.005), Boxing & Wrestling (ANOVA, *p* < 0.002) and Pinning (ANOVA, *p* < 0.04), with LPS reducing play in males but not in females. *: Planned comparisons confirmed that LPS Males showed less Total Play (Fisher’s PLSD, p < 0.006) and Boxing & Wrestling (Fisher’s PLSD, p < 0.0008) than did Saline Males. #: Saline Males had higher scores than did Saline Females for Total Play (Fisher’s PLSD, p < 0.05) and Boxing & Wrestling (Fisher’s PLSD, p < 0.03). In treatment-mixed pairs, no significant interactions of treatment X sex were found. Planned comparison, however, suggested that LPS reduced Pouncing in males (Fisher’s PLSD, p < 0.04) but not in females. By definition, when one animal in a pair shows Boxing & Wrestling, so does the other. For that reason, we show only one differently shaded bar for LPS and Saline animals for Boxing & Wrestling rather than two identically sized bars for males and females in mixed-treatment pairs.

In total, behavior was tested in twentyeight male and thirtyone female pups derived from seven saline-treated and five LPS-treated litters. As the data were averaged by litter, n = 7 per group was used for saline-treated individuals and n = 5 per group for LPS-treated individuals in the statistical analysis. In treatment-matched pairs, maternal LPS injection significantly reduced Total Play in males but not in females, which was reflected in a significant sex X treatment interaction (Figure 1; F(1,20) = 10.19, p < 0.005). Consistent with previous findings [20,21], Saline Males played significantly more than Saline Females (Figure 1; planned comparison, p < 0.05). To determine whether the LPS effect was evident in specific play behaviors, we analyzed Boxing & Wrestling, Pouncing, and Pinning separately. Both Boxing & Wrestling as well as Pinning exhibited the same significant sex X treatment interaction seen in Total Play (Figure 1; F(1,20) = 14.45, p < 0.002 for Boxing and Wrestling and F(1,20) = 5.37, p < 0.04, for Pinning). No main effects or interactions were found for Pouncing (Figure 1). As males and females have different developmental trajectories, with females reaching puberty earlier, differences in maturation could contribute to sex differences in LPS effects. However, females showed no LPS effects at any of the ages that behavior was measured.

As social play is dyadic, potential effects of LPS in an animal may also depend on treatment of its partner. Therefore, we tested LPS effects in mixed-treatment pairs as well (treatment-mixed pairs). Also in this case, group means exhibited a similar trend. For example, LPS marginally reduced Pouncing and Pinning in males (planned comparisons, p < 0.04 and p = 0.06 for Pouncing and Pinning, respectively) but not in females (Figure 1). Treatment differences in Total Play, however, were blunted and not significant (Figure 1). Given that Boxing & Wrestling is by definition displayed by both animals of the pair at the same time (indicated by the gray columns in Figure 1), and given also that it comprises a large proportion of Total Play, it may have masked an LPS effect on Total Play in mixed pairs. Matched and mixed pairs also differed in their familiarity with the play partner, with matched animals being paired with a cage mate and mixed animals with an unfamiliar animal. It is possible that differences between LPS and Saline Males would have been more, or perhaps less pronounced if treatment-matched animals would have been tested with unfamiliar cage mates.

Sex differences in LPS effects on play behavior were clearest in matched pairs. In treatment-mixed pairs, fewer sex differences were found, perhaps because the LPS Males might have been less inclined to play, thereby bringing down the overall score of their Saline Male partners. In support, in mixed pairs, Pinning was marginally higher in Saline than in LPS Males (Figure 1 planned comparison, p = 0.06).

### LPS effect on AVP mRNA expression

**Figure 2:**
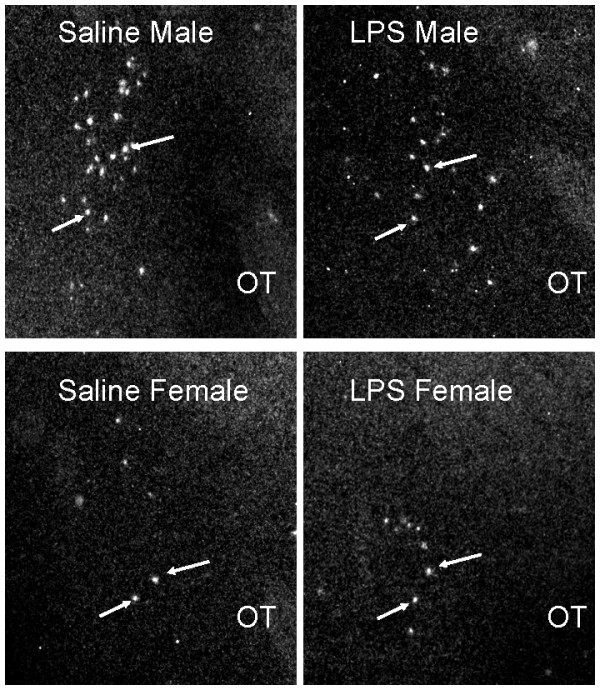
**AVP mRNA expression in the medial amygdaloid nucleus (MeA).** Photomicrographs show sections processed with *in situ* hybridization for AVP mRNA. Arrows indicate individual cells labeled for AVP mRNA; ot: optic tract.

**Figure 3:**
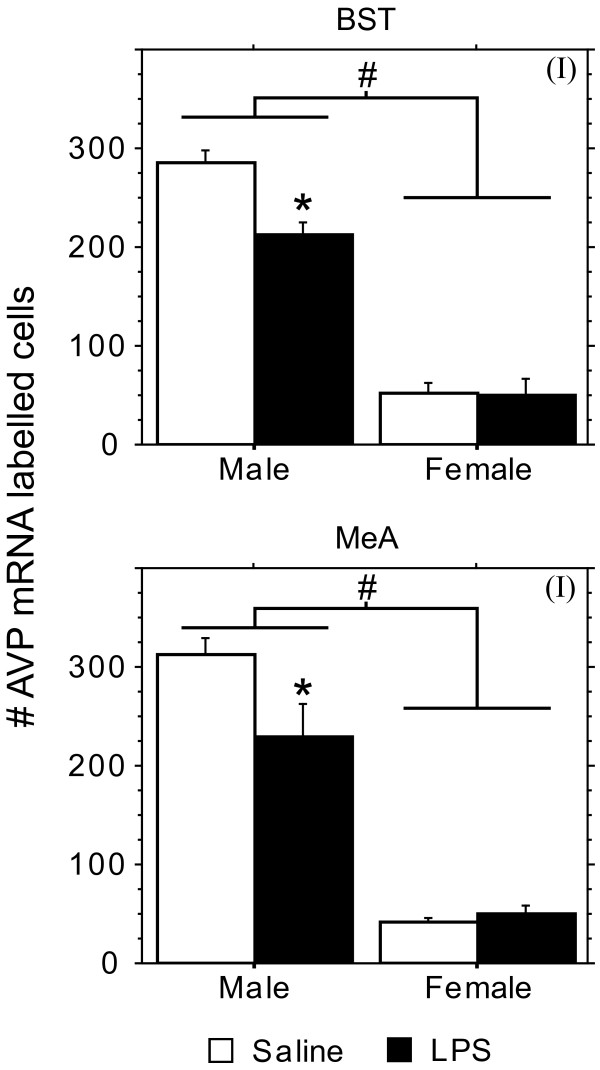
**Effect of prenatal LPS on vasopressin expression in the MeA and BST.** Means (+ SEM) of the number of AVP mRNA-labeled cells in the MeA and BST. #: Overall, males had significantly more AVP mRNA-labeled cells than did females (ANOVA, p < 0.0001 for MeA as well as BST). (I): There was a significant sex X treatment interaction: LPS treatment reduced the number of cells in the MeA and BST in males but not in females (ANOVA, p < 0.04 and p < 0.03 for MeA and BST, respectively). *: Planned comparison showed that LPS males had significantly fewer labeled cells than did Saline Males (Fisher’s PLSD, p < 0.02 and p < 0.005 for the MeA and BST, respectively).

**Figure 4:**
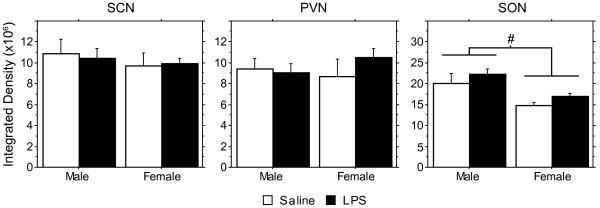
**Lack of effect of prenatal LPS on vasopressin expression in the SCN, PVN, and SON.** Means (+ SEM) of the integrated density of AVP mRNA labeling in the SCN, PVN, and SON. Although there was no effect of LPS treatment, overall, males showed higher integrated density in the SON than did females (ANOVA, p < 0.008).

In total, thirty brains were processed for AVP mRNA expression from male and female pups derived from three LPS-treated and three saline-treated litters. Due to poor histology some material could not be analyzed: 1 brain was excluded for the MeA, 4 for the BNST and SCN, 5 for the SON, and 6 for the PVN. As the data were averaged by litter, n = 3 per group was used for statistical analysis. Significant effects of LPS treatment on AVP mRNA expression were only found in the MeA and BST (Figure 2). Confirming the literature [22], juvenile males showed more AVP mRNA-expressing cells in the MeA and BST than females, (Figure 3; F(1,8) = 146.98, p < 0.0001; F(1,8) = 236.2, p < 0.0001 for MeA and BST, respectively). LPS treatment reduced AVP expression in males but not in females, thereby causing significant treatment X sex interactions in the MeA and BST (Figure 3; F(1,8) = 6.11, p < 0.04; F(1,8) = 7.45, p < 0.03 for MeA and BST, respectively). These sex-specific effects of LPS were restricted to the BST and MeA, as there were no LPS effects on AVP mRNA expression in the SON, PVN, or SCN (Figure 4). In accord with what has been reported for the size of the SON and its AVP neurons in 60-day old rats [23], we found that volume of the area expressing AVP mRNA and integrated density of AVP mRNA expression in the SON are larger in males than in females (Figure 4; F(1,8) = 12.57, p < 0.008 and F(1,8) = 12.86, p < 0.008 for volume and integrated density, respectively).

### Prenatal immune activation does not impact sexual differentiation of the SDN-POA

**Figure 5:**
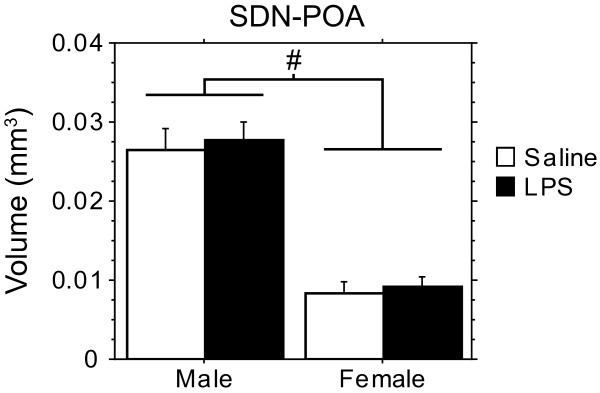
**Effect of prenatal LPS on the volume of the SDN-POA.** Means (+ SEM) of the volume of the SDN-POA. #: The volume of the SDN-POA is significantly larger in males than in females (ANOVA: p < 0.0001). LPS did not affect the volume of the SDN-POA.

In total, thirtytwo brains were thionin-stained from male and female pups derived from four saline and three LPS-treated litters. As the data were averaged by litter, n = 4 (saline) and n = 3 (LPS) per group was used for statistical analysis. As in adult animals [18] the SDN-POA was about three times larger in males than in females (Figure 5; F(1,10) = 78.92, p < 0.0001). LPS treatment had no effect on this difference.

## Discussion

We found that treating rats with LPS on day 15 of pregnancy reduced social play of male but not of female offspring. Only one other study reports that LPS exposure on day 9.5 of pregnancy reduces play in male offspring; no females were studied, and no effects were found on gross morphology of the brain [24]. We found that, in addition to social play, LPS reduced AVP expression in the BST and MeA, again in male but not in female offspring. Therefore, our findings suggest that the often-made observation that males are more vulnerable to prenatal stress than females [25,26] can be extended to the neural substrate underlying social behavior in juvenile animals.

Social play as well as AVP expression in the BST and MeA are more prominent in male than in female juveniles [20,22]. As LPS treatment reduced social play and AVP expression in males only, LPS treatment may have interfered with general mechanisms of sexual differentiation. For example, malnutrition or environmental stress in the last week of pregnancy reduces differentiation of sexual behavior and SDN-POA volume [27]. A stress-linked reduction in fetal activity of testosterone may contribute to this effect [28,29]. As sexual differentiation of play behavior and AVP expression depends on higher levels of testosterone in males [30-33], LPS may have inhibited masculinizing effects of testosterone. Our data, however, argue against a general effect of LPS on sexual differentiation, as the volume of the SDN-POA was unaffected by LPS treatment.

There are several other possible explanations as to why LPS treatment affected AVP expression in males but not in females in the present study. Humoral factors generated as a result of LPS treatment may have differential access to male and female fetuses. For example, stress early in pregnancy significantly changes the expression of genes implicated in the hypoxic response, cell differentiation, and metabolism in male but not in female placentas [34]. Immune challenges may have similar dimorphic effects on the placenta, thereby possibly differentially affecting the exchange of nutrients, metabolic waste products, and signaling molecules across the placental barrier.

LPS treatment may also have made AVP cells less sensitive to the masculinizing effects of gonadal steroids postnatally. Higher levels of testosterone found in males increase the probability that developing neurons in the BST and MeA commit to a vasopressinergic phenotype [35,36]. Given that LPS effects were only significant in males, LPS may have interfered with this differentiating step. Interestingly, sexual differentiation of specific brain areas and behaviors uses components of signal transduction pathways that are common to inflammatory processes [37-39]. As AVP cells in the BST are affected by inflammatory processes in adult animals [40], there may be cross-talk between sexual differentiation and immune signaling pathways during their development as well.

Several lines of evidence suggest that the LPS-induced reduction in play behavior and in AVP mRNA expression in the MeA are causally related. AVP has been implicated in the control of social behavior [6,41]. Moreover, injecting an AVP receptor antagonist intracerebroventricularly reduces play behavior in males [8]. Furthermore, systemic testosterone can masculinize play behavior as well as AVP expression in the MeA and BST by acting on androgen rather than estrogen receptors [30,33], and intracranial testosterone implants placed specifically into the amygdala masculinize social play [31]. It is not yet known whether AVP treatment can reverse the reduction in play behavior in LPS Males.

The LPS-induced reduction in AVP mRNA was specific to the BST and MeA, as levels did not change it in the SON, PVN, or SCN. Differences in birth date of AVP neurons may contribute to differences in LPS effects on various AVP-expressing brain regions in this study. AVP neurons in the BST and MeA are born on embryonic days 11 and 12 [42,43] and therefore all of these cells could be affected by LPS treatment on embryonic day 15. In contrast, SCN cells are born on embryonic days 14–17 [44], which is, by and large, at or after the LPS treatment given in this paper. Differences in developmental trajectory may also shelter the PVN and SON, as neurons in these nuclei are born on embryonic days 12–18. Thus only a fraction of the cells are born by the time LPS was administered [44].

BST and MeA cells appear to be responsive to immune challenges in adulthood as well. In adult rats, LPS treatment acutely increases AVP release in the ventral septal area [45], a projection area of AVP neurons of the BST and MeA [46], and treatment with the pro-inflammatory cytokine interleukin-1beta, which is released upon exposure to LPS, increases the firing rate of BST and MeA neurons [47]. These effects have been linked to AVP’s role in fever abatement [40]. We propose that LPS treatment early in life may activate these same neurons, thereby permanently changing their impact on physiology and behavior. A relevant example of such programming is found in the administration of LPS early postnatally, which permanently alters the fever response, interestingly, more so in males than in females [48].

As of yet it is unclear what molecular mechanisms underlie LPS-induced changes in AVP expression and play behavior. Most likely, early immune activation altered the fate of a number of cells, perhaps by changing epigenetic regulation of AVP gene expression. Such long-term changes have been shown for the PVN, where early life stress increases AVP expression while reducing methylation of CpG sites of a chromosomal region that controls AVP expression [49]. Related epigenetic changes might underlie the effects of prenatal immune activation on social behaviors reported here.

In addition to LPS treatment directly affecting fetal development, it may have altered maternal behavior, and thereby development. Several studies suggest that this is not likely. For example, dams injected with the same dose of LPS used in this study, but on embryonic day 15 as well as day 16, did not show changes in parental care [50]. In addition, stress during fetal development changes social behavior and expression of oxytocin mRNA in the PVN of adult male rats, irrespective of whether they were raised by stressed or unstressed dams [51]. However, given the role of maternal care in male sexual differentiation [52], the possibility remains that changes in maternal behavior may have mediated or masked potential effects of prenatal LPS.

## Conclusions

This study demonstrated that LPS treatment on day 15 of pregnancy affects play behavior in male but not in female offspring. Likewise, LPS treatment reduces AVP expression in male but not in female offspring, specifically in AVP-expressing nuclei that have been implicated in social behavior. Conditions that activate the immune response during pregnancy increase the frequency of diagnoses for autism, schizophrenia, and depression [1,2]. Interestingly, all these disorders are sexually dimorphic with respect to onset, course, and incidence. For example, schizophrenia is more common in men [53], and autism is more common in boys [54]. Sex differences in vulnerability to stress may be due to differences in neural systems that modulate social behavior, such as the AVP system. Indeed, converging evidence suggests that AVP may be involved in social disorders such as autism [55,56] as well as in normal aspects of human social behavior [57,58]. The evidence for involvement of AVP in social behaviors such as social recognition, parental, and aggressive behaviors is even stronger in laboratory animals [7,41,59,60]. Interestingly, AVP influences social behavior differently in male and female rodents [61]. The same may be true in humans as well [57]. If so, developmental perturbation of AVP innervation is prone to affect one sex more than the other. In that regard, the influence of LPS on the development of social play may be a good model for understanding factors that contribute to sex differences in social psychopathology.

## Abbreviations

AVP, Arginine vasopressin; LPS, Lipopolysaccharide; MeA, Medial amygdala; SON, Supraoptic nucleus; PVN, Paraventricular nucleus; BST, Bed nucleus of the stria terminalis; SCN, Suprachiasmatic nucleus; SDN-POA, Sexually dimorphic nucleus of the preoptic area; mRNA, Messenger RNA.

## Competing interests

All authors declare that there are no conflicts of interests

## Authors’ contributions

PVT and GJD conceived of and designed the study. PVT, AHV, RB, and SI treated the animals and video-taped their behavior. PVT, AHV, MJP, and SI developed and participated in the scoring of video-recorded behaviors. PVT, RB, SI, and GJD processed brains histologically and performed microscopic analysis of brain tissue. PVT, MJP, and GJD performed the statistical analysis. PVT, GJD, and MJP drafted the manuscript, and all authors read, made suggestions for changes, and approved the manuscript.
